# Reliability and validity of motion analysis in children treated for congenital clubfoot according to the Clubfoot Assessment Protocol (CAP) using inexperienced assessors

**DOI:** 10.1186/1756-0500-2-103

**Published:** 2009-06-12

**Authors:** Hanneke Andriesse, Gunnar Hägglund, Per-Erik Isberg

**Affiliations:** 1 Lund University Hospital – Lund University Faculty of Medicine, Department of Orthopedics, Lund University Hospital, SE-22185, Lund, Sweden; 2 Department of Statistics – Lund University School of Economics and Management, Statistical Institution, Lund University, Scheelevägen 15B, Box 743, SE-22007, Lund, Sweden

## Abstract

**Background:**

The Clubfoot Assessment Protocol (CAP) was developed for follow-up of children treated for clubfoot. The objective of this study was to analyze reliability and validity of the six items used in the domain CAPMotion Quality using inexperienced assessors.

**Findings:**

Four raters (two paediatric orthopaedic surgeons, two senior physiotherapists) used the CAP scores to analyze, on two different occasions, 11 videotapes containing standardized recordings of motion activity according to the domain CAPMotion Quality These results were compared to a criterion (two raters, well experienced CAP assessors) for validity and for checking for learning effect.

Weighted kappa statistics, exact percentage observer agreement (Po), percentage observer agreement including one level difference (Po-1) and amount of scoring scales defined how reliability was to be interpreted. Inter- and intra rater differences were calculated using median and inter quartile ranges (IQR) on item level and mean and limits of agreement on domain level.

Inter-rater reliability varied between fair and moderate (kappa) and had a mean agreement of 48/88% (Po/Po-1). Intra -rater reliability varied between moderate to good with a mean agreement of 63/96%. The intra- and inter-rater differences in the present study were generally small both on item (0.00) and domain level (-1.10). There was exact agreement of 51% and Po-1 of 91% of the six items with the criterion. No learning effect was found.

**Conclusion:**

The CAPMotion quality can be used by inexperienced assessors with sufficient reliability in daily clinical practice and showed acceptable accuracy compared to the criterion.

## Background

The Clubfoot Assessment Protocol (CAP) [1,2] (Table 1) was developed for follow-up of children treated for congenital clubfoot. Twenty items divided over four domains (Mobility, Muscle function, Morphology and Motion Quality) form the CAP. Most previous instruments for evaluation of children with clubfoot, such as the International Clubfoot Study Group evaluation system (ISGC) [3] and the Laaveg-Ponseti [4], do not include items concerning the child's quality of motion, as in walking or running.

**Table 1:** A summary of the Clubfoot Assessment Protocol (CAP)

Subgroup	Items	Scores (Worst to best)
Mobility I	Ankle dorsal extension Ankle plantar flexion Heel varus/valgus Eversion/inversion Forefoot adduction/abduction	Item level:0,1,2,3 and 4 (Total sub score: 0–20)
Mobility II	Length of toe flexors: M. flexor digiti longus M flexor digiti hallucis	Item: 0, 2 and 4. (Total sub score: 0–8)
Muscle function	Strength of foot eversion: M. peronus longus M.extensor digiti longus	Item: 0, 2 and 4. (Totalsub score: 0–8)
Morphology	Tibial torsion, Heel position Forefoot position Cavus or planus.	Item: 0, 2 and 4. (Total sub score: 0–16)
Motion quality I	Walking Running Toe walking Heel walking	Item: 0, 1, 2, 3 and 4. (Total sub score: 0–16)
Motion quality II	One-leg balance One-leg hop	Item: 0, 1, 2, 3 and 4. (Total sub score: 0–8)

0 = cannot/++ poor, 1 = very deviant/+ poor, 2 = deviant/poor, 3 = slightly deviant/± poor and 4 = within normal.

Completed with questions about experience of stiffness, pain, shoe problems, participation in sports, problems with keeping up with peers or other specific problems in daily life.

The CAP has in previous studies shown good reliability, validity and sensitivity for change in the four domains Mobility, Muscle function, Morphology and Motion quality with experienced assessors. [1,2]

The objective of this study was to analyze the intra-and inter rater reliability of the items used in the domain Motion Quality of the CAP and their validity, with inexperienced CAP assessors.

## Methods

### CAPMotion Quality

This domain contains six items; running, walking, toe walking, heel walking, one-leg hop and one-leg balance (see additional file 1). At the age of four years children are normally expected to be able to perform all six items. In the appendix the scoring distribution and criteria are described. The scoring has been divided systematically in proportion to what is regarded as normal variation and its supposed impact on the child's physical function. Assessment is done in relation to the child's age.

### Patients

Video recordings of eleven children treated for clubfoot and with varying severity and outcome results, were selected from the archives of our clubfoot clinic. The tapes contained standardized recordings of motion activity according to the domain CAP**Motion Quality**. The median age was 5. 5 years (range 4 – 7 years). Gender distribution was three girls and eight boys. Five children had unilateral clubfoot and six bilateral. All families gave their informed consent for the use of the video films.

### Raters

Four raters were selected according to the criteria having worked within pediatric orthopedics at least seven years including experience with children with clubfoot. Two raters were pediatric orthopedic surgeons and two were senior physiotherapists. None of the raters had previous experiences with the CAP system.

Two raters well experienced with CAP, one physiotherapist and developer of the CAP (HA) and one pediatric orthopedic surgeon (GH), defined the most correct score for each child's item performance.

### Video recording

The recording procedure was standardized and comparable with the situation in a daily clinical environment. The children were recorded from a frontal and posterior view while moving along a 10 meter pathway. The camera was positioned on one meters height and two meter from the beginning of the pathway. The children wore t-shirts, shorts or underwear and were barefoot. The children were asked if they wanted to start with walking or running. All children started with running followed by walking, toe walking, heel walking, one-leg hop and on-leg stance. Recordings were made of each performance as much as necessary to be able to make an assessment comparable with real life. Each video sequence lasted about 4 minutes.

### Rating procedures

All four raters received three weeks before the first assessment session the CAP**Motion Quality **manual with the items criteria and a copy of the protocol form to be used during the rating session (see additional file 1). They were asked to study the manual and scoring system and use this information during the assessment sessions.

Each rater assessed individually all 11 video recorded children twice within an interval of 4 to 6 weeks.

An introduction was given prior to each assessment session explaining the testing procedure; 1) After each video recording a half minute pause was given. A short brake was made after the fifth video. 2) No possibilities were given to stop the video or to assess the recordings in slow motion. 3) Before each new video sequence the raters received only information about the child's age and gender. 4) Both left and right side should be rated. As a training session, the raters viewed and at the same time rated a videotaped recording of a child without a disability and a child treated for congenital clubfoot. Total testing time was approximately one hour and 15 minutes.

The two experienced assessors (HA and GH) analyzed and discussed the same videos at one meeting and defined the most correct rating for each side and each child. This was done before the first assessment of the four raters.

### Data analyses

Both legs were rated and used as individual ratings in the statistical analyses.

Inter – and intra tester reliability was calculated using the weighted kappa (*k*) statistics [1,2] together with its 95% confidence intervals. For the inter-rater testing the assessments from the first sessions were used. According to Altman [5] the kappa values are to be interpreted as follows: < 0.20 as poor agreement, 0.21 – 0.40, as fair, 0.41 – 0.60 as moderate, 0.61 – 0.80 as good and > 0.80 as very good. Exact observed percentage agreement (Po) and percentage agreement including one level difference (= Po-1) were calculated as kappa values can become unstable under certain conditions, e.g. with limited distribution of cell frequency [6-8]. As the CAP**Motion quality **domain exists out of five scoring possibilities we regarded a Po ≥ 50% or a Po-1 ≥ 80% as good.

Good item reliability was considered when more than halve of the assessment pairs had kappa's values higher than 0.60 (= good) and/or a good percentage agreement. Sufficient item reliability was considered when the kappa values ranged between 0.41–0.60 (fair to moderate) for more than halve of the inter-intra ratings and/or had good percentage agreement.

The median differences and inter quartile ranges (IQR) for each item (ordinal data) and the mean difference and its limits of agreement (LOA) (interval data) for the domain motion quality for the inter-and intrarater were calculated. [5]

For evaluating if there was a learning effect between the first and second session, the Po and Po-1 assessed with the criterion, were used. A difference of more than 10% was set as level for a real difference.

## Results

### Inter-rater reliability

The item inter-rater reliability between the four raters is presented in Table 2 and 3. In general, item inter-rater reliability according to kappa statistics varied between fair and moderate and had a mean item Po/Po-1 of 48 and 88%. The median differences between all ratings (n = 132) (Inter Quartile Range) for item running to item 1-leg hop was 0.00 (-1.0–0.0), 0.0 (-1.0–0.0), 0.0 (-1.0–0.0), -1.0 (-2.0–0-0), 0.0 (0.0–1.0), 0.0 (-1.0–0.0) respectively. The mean difference and the Limits of Agreement (LOA) (n = 776) for the domain Motion Quality was -1.10 (-1.86–1.66)

**Table 2:** Inter-rater reliability between the four raters at session one

**Item**	**A-B**		**C-D**	
	
	**k**	Po/Po-1	**k**	Po/Po-1
Running *	0.30 (0.06–0.54)	36/77	**0.45 **(0.21–0.69)	**68/100**
Walking *	0.37 (0.12–0.62)	46/**91**	0.32(0.07–0.56)	**55/100**
**Toe-walking**	0.30(0.07–0.54)	36/73	**0.62 **(0.40–0.83)	**65/100**
**Heelwalking**	0.12 (-.30–0.28)	23/59	**0.60 **(0.41–0.81)	**62/100**
1-leg stance*	**0.57 **(0.37–0.77)	**55/91**	**0.61 **(0.38–0.84)	46/**86**
1- leg hop *	**0.49 **(0.26–0.71)	27/**86**	**0.68 **(0.50–0.87)	45/**95**

**Table 3:** Inter-rater reliability between the four raters at session one

	**A-C**		**B-C**		**A-D**		**B-D**	
	
	**k**	Po/Po-1	**k**	Po/Po-1	**k**	Po/Po-**1**	**k**	Po/Po-1
Running *	0.25 (0.08–0.42)	41/**91**	0.35(0.14–9.56)	**50/91**	**0.44 **(0.18–0.71)	**64/91**	**0.46 **(0.22–0.70)	**54/100**
Walking *	0.06 (-0.1–0.26)	36/77	**0.52 **(0.26–0.77)	**64/96**	**0.42 **(0.15–0.68)	**55/96**	**0.54 **(0.27–0.82)	**64/96**
**Toe-walking**	0.38 (0.17–0.59)	41/77	0.38(0.07–0.69)	41/**100**	0.35 (0.11–0.59)	35/**81**	**0.72 **(0.47–0.98)	**75/100**
**Heelwalking**	0.38 (0.15–0.60)	32/77	0.31 (0.05–0.56)	**50/86**	**0.57 **(0.33–0.81)	**62/81**	0.29 (0.05–0.53)	48/67
1-leg stance*	**0.53**(0.29–0.76)	**55/82**	**0.45 **(0.24–0.66)	41/**82**	0.15 (-0.16–0.4)	27/72	0.37 (0.11–0.62)	41/**81**
1- leg hop *	**0.79 **(0.63–0.94)	**68/100**	**0.51 **(0.29–0.74)	36/**91**	**0.57 **(0.37 0.77)	45/**95**	**0.42 **(0.16–0.68)	35/**90**

• * = sufficient item reliability. Bold numbers = above defined reliability cut off points

• **K **= kappa (95%CI), Po/Po-1 = Exact observed percentage agreement/percentage agreement including one level difference

According to the reliability criteria four out of six items showed sufficient inter-rater reliability. The items toe-and heel walking showed overall problems with sufficient assessment agreement.

### Intra-rater reliability

The median intra-rater reliability for the individual raters is presented in Table 4. In general item intra reliability varied between moderate to good and had a mean item Po/Po-1 of 63/96%. The median differences for all raters (n = 88) (Inter Quartile Range) from item running to item1-leg hop was 0.00 (0.0-0.0), 0.0 (0.0-0.0), 0.0 (0.0-0.0), -1.0 (0.0-0.0), 0.0 (-1.0–0.0), 0.0 (0.0-0.0). The mean difference and the Limits of Agreement, (LOA) (n = 522) for the domain Motion Quality was -0.04 (-1.34–1.27)

**Table 4:** Intra-rater reliability for each rater

Item	A		B		C		D	
	
	**k**	Po/Po-1	**k**	Po/Po-1	**k**	Po/Po-1	**k**	Po/Po-1
Running*	**0.45 **(0.21–0.69)	**50/96**	**0.61 **(0.37–0.85)	**82/96**	**0.55 **(0.23–0.86)	**77/100**	**0.55 **(0.24–0.86)	**72/100**
Walking*	0.32 (0.07–0.56)	46/**96**	**0.68 **(0.45–0.90)	**64/86**	0.22 (-0.03–0.49)	**50/100**	**0.42 **(0.09–0.76)	**64/96**
Toe-walking**	**0.62 **(0.40–0.83)	**64/91**	**0.72 **(0.48–0.96)	**73/100**	**0.67 **(0.48–0.87)	**68/100**	**0.76 **(0.55–0.97)	**79/100**
Heel-walking**	**0.60 **(0.41–0.81)	**55/91**	0.37 (0.05–0.69)	46/**91**	**0.80 **(0.62–0.99)	**82/100**	**0.66 **(0.44–0.89)	**67/100**
1- leg stance*	**0.61 **(0.38–0.84)	**59/82**	**0.58 **(0.31–0.86)	**55/96**	**0.52 **(0.26–0.78)	**50/91**	**0.56 **(0.27–0.84)	**59/100**
1- leg hop**	**0.68 **(0.50–0.87)	**50/86**	**0.71 **(0.49–0.93)	**68/96**	**0.81 **(0.68–0.95)	**73/100**	**0.64 **(0.43–0.84)	**55/100**

• * = sufficient item reliability. ** = good item reliability. Bold numbers = above defined reliability cut off points

• **K **= kappa (95%), Po/Po-1 = Exact observed percentage agreement/percentage agreement including one level difference

Three items showed good reliability and three showed sufficient intra-rater agreement.

### Learning development and validity with the criterion

**Figure 1:**
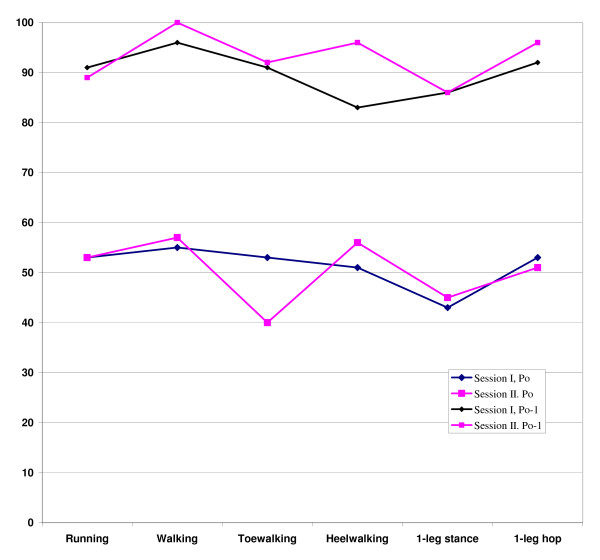
**The exact mean percentage agreement (Po) and the within one level disagreement (Po-1) between the four observers and the criterion for the six items at testing session I and II**.

No general improvement was seen between the first and second session regarding the exact observed mean percentage agreement for all items (Figure 1). Item toe walking showed decreased agreement (from 53 to 40%) Also when including one category difference for the observed percentage agreement, no improvement occurred between the first and second session except for item heel walking (83 to 96%). These results also showed an exact agreement of 51% and Po-1 of 91% of the six items with the criterion.

## Discussion

This is, to our knowledge, the first study focusing on reliability on assessing different activity performances in children born with clubfoot in a situation comparable with a daily clinical setting. The inter-rater reliability for four out of six items from the CAP**Motion quality **showed sufficient reliability. The items toe-and heel-walking showed fair reliability. The observers' intra-rater reliability showed reliability between good to sufficient for all items. Inter- and intra rater score differences on item and domain level were relatively small. No clear learning effects were found between the first and second session.

Three-dimensional gait analyses (3DGA) are commonly advocated as the golden standard within gait analysis. In our study these computerized motion analyses were not useful for validation of our items as they are not (yet) obtainable. The exact agreement of 51% with our criterion, the five scorings possibilities and the Po-1 agreement of 91% shows evidence for a valid assessment system. It will be interesting to study how more experience of the CAP system or a CAP course can increase the validity and reliability.

### Methodological issues

It is impossible to actual calculate the true reliability of an instrument. Many internal factors such as sample size, amount of scoring possibilities, statistical method, and external factors such as assessment procedure and shifting performance of the object under observation, can influence the outcome of studies on reliability. In studies with young children these external factors can be very difficult to keep stable. We tried to control the external factors by using video recordings which resembled as much as possible the daily clinical situation. This made it possible for several raters to assess the same phenomenon.

Strictly methodologically it is not correct to use both legs of the same child as individual ratings as they can be dependent of each other. This can be a significant problem in treatment outcome studies. In the present study, however, we think this is of minor importance as the aim was to study the reliability of assessors when they have to assess both legs similar to the normal clinical situation.

Defining the cut off points for the percentage agreement is arbitrarily. A concordance of 75–80% with two possibilities is commonly used. We think that our cut off point with 50% for the exact percentage agreement with a 5-point scale is acceptable. We also checked the score differences for information on the clinical implication of the reliability.

We tried to integrate different information on the instruments behavior with inter-and intra rater testing trying to create an as truthful picture as possible.

Wren et al [9] found in their reliability study of visual gait assessments in children with pathologic gait no statistically significant differences in reliability between "live", full speed and slow speed video. In some cases though, slow motion video improved agreement of observational assessments. Brunnekeef et al [10] concluded that structured visual gait observation by use of a gait analysis form was moderately reliable in patients with orthopedic disorders. Clinical experience appeared to increase the reliability of visual gait analysis.

The observers in the present study explained difficulties in not having control over the assessment situation. The raters were not allowed to stop, rewind or see the recording in slow motion. This might have had a negative influence on our reliability and validity results.

Knowledge about the score differences between observers is important as this has to be incorporated in studies on responsiveness. The intra- and inter-rater measurement errors in the present study were small both on item and domain level. Celebi et al. [11] found mean difference scores of 0.17, 0.63 and 0.80 (LOA around – 2.00 to 3.00) between three experienced observers for their functional domain of the International Clubfoot Study Group evaluation system (ISGC) [3]. This domain has a total score of 36 and uses 2 -or 3 point scales. Our result; -1.10 (-1.86–1.66) for the CAP**Motionquality **with a total score of 24 and a 5 point scale is in comparison very promising.

Fewer scoring levels would probably increase the reliability for the CAP**Motionquality **items, but decrease the sensitivity for differences. An instrument with higher sensitivity is clinically more informative. More scoring possibilities also demands the administrator to more critically assess an observation and decide which scoring is the most correct. These situations can have a learning effect and with time increases the quality and reliability of assessments.

## Conclusion

We conclude that the CAP**Motion quality **can be used with good reliability and validity in daily clinical practice. When different observers are used, and within research, it is recommended to check the inter-rater reliability and calculate the scores differences on item or domain level.

## Competing interests

The authors declare that they have no competing interests.

## Authors' contributions

HA and GH designed and collected the data. HA and P-EI analyzed, interpreted and revised the data and manuscript. HA drafted the manuscript. GH and P-EI revised the manuscript.

## Supplementary Material

Additional file 1 **Appendix**. Summary of the Clubfoot Assessment Protocol (CAP version 1.1) domain motion quality. Click here for file
