# Supplementary material for: Reliability and validity of motion analysis in children treated for congenital clubfoot according to the Clubfoot Assessment Protocol (CAP) using inexperienced assessors
Source: BMC Res Notes. 2009 Jun 12;2:103. doi: 10.1186/1756-0500-2-103 (PMC2705375; doi:10.1186/1756-0500-2-103)
Supplement: Additional file 1 — Appendix. Summary of the Clubfoot Assessment Protocol (CAP version 1.1) domain motion quality. [file 1756-0500-2-103-S1.doc]

**Appendix**

Summary of the Clubfoot Assessment Protocol (CAP version 1.1) domain motion quality.

y = years of age starting for testing

Rating **0 1 2 3 4**

**Motion quality**

**I.**

15. Running 2y cannot + deviant deviant slightly deviant normal

16. Walking 2y cannot +deviant deviant slightly deviant normal

17. Toe walking 3y cannot +deviant deviant slightly deviant normal

18. Heel walking 3y cannot +deviant deviant slightly deviant normal

**II.**

19. One-leg stand 4y cannot +deviant deviant slightly deviant normal

20. One-leg hop 4y cannot +deviant deviant slightly deviant normal

**CAPmotion quality: Quality of basic motor performance.**

Movement quality focuses on the child’s ability to perform an activity in an age appropriate manner, which includes sufficient and efficient power generation, joint kinematics and body balance.

The intention of this category of the CAP is to try to provide structure as well as to standardise the observation of various activities which after literature study, clinical experience and colleagues discussion where thought to be valid for a profile of the child’s functional ability.

**Rating:** Each activity is rated between 0-4 points (5 level scales). For each rating a description of criteria are provided. The amount of points is related to the impact it is expected to have on the child’s activity- participation level in accordance with the classifications levels used in the ICF.

The assessment is to be done in a surrounding where the child feels at ease. The younger child will need to be stimulated to perform the movements we wish to observe. Playing situation can provide us with rich information. Even observation of older children performing non-specific tasks, such as playing a ball, provides us with information.

If problems with performance are thought to be caused by other factors other than decreased mobility and periphery muscle function such as hypo/hypertoni, hyperactivity, concentration problems,” clumsiness and/or obesity this should be noted separately.

It should also be noted that the information gathered from the clinical assessment should be disregarded while assessing the activity. It is very easy to be influenced and prejudiced, altering ones judgement. The aim is to assess the quality on how the activity is performed and not to analyse it.

Testing environment: Normal clinical situation with a corridor approximately 10 meters in length. Parent standing on one side and examiner on the other side.

Testing procedure: A good way of starting the performance test is to let the child decide if he/she wants to begin with running or walking. Being able to have some control over the situation makes the child feel at ease.

**1. Running.**

Remember that some children have a tendency to run more on the forefoot, which can be a normal variation in running.

Observe in the same way as described for walking.

Instruction: Run as fast as you can to your Mother/Father and than back again to me. Ready steady go…..

**4 points, normal:**

Nice flow, no compensation on knee/hip level, good foot position during the whole stride. Straight-line.

**3 points, slightly deviant:**

1. Clear in toeing without lateral loading and /or varus, normal progression over the foot.

Or

1. Tendency to in toeing and lateral loading.

**2 points, deviant:**

In toeing and lateral loading, tendency for “rolling” over the lateral foot border creating a feeling that the child is bow-legged

Or

1. IC lays more lateral and anteriorly causing a short step length, increased hyperextension on knee level.

Or

1. Increased knee flexion in stance, clear decreased push-off power, difficult to increase speed.

**1 point, very deviant:**

Same as 2 points though together with clearly increased pelvic movement. Energy consuming. Difficult keeping a straight progression line.

**0 points:**

Cannot accomplish the task.

**2. Walking**

Observe the child’s ability to walk straight, step length, IC (= initial contact of heel), knee/hip movement (hyperextension/flexion); roll over the foot (loading), in toeing (varus-adductus foot, tibia inward rotation, hip-ante version).

Instruction: Walk straight towards your Mother/Father and (once there) back to me (= the examiner).

**4 points, normal:**

Smooth pattern, normal initial heel contact (IC), good progression over the ankle, normal knee flexion/extension pattern, hip stabilisation, normal foot progression angle. A very slight in or out toeing.

**3 points, slightly deviant:**

1. Only in toeing or out toeing with normal IC, loading and progression over the ankle/ foot.

Or

1. A tendency to in toeing together with a tendency for tipping laterally. Normal knee/ hip movement.

**2 points, deviant:**

1. A clear in toeing with lateral loading and/ or varus of the heel.

Or

1. Normal/nearly normal foot progression though compensation on knee/hip level such as genu varum, hyperextension in knee in stance phase. Short stride length, no clear initial heel contact.

Or

1. Increased ankle dorsal flexion and / or knee flexion in stance

**1 point, very deviant:**

1. No IC and/ or significant lateral loading hyperextension /instability on knee level. Increased hip flexion and increased pelvic rotation.

Or

1. Highly increased in toeing with clear compensation mechanism on knee/hip level such as increased hip outward rotation

The movement looks energy consuming. Difficulties at keeping a straight walking path.

**0 points:**

Cannot accomplish the task.

**3. Toe walking.**

Observe how much maximal plantar flexion the child is able to achieve. Look at stabilisation around knee and ankle and position/ alignment in relation to upper body. Does the child manage to keep good maximum height or does he /she “start dropping” the heel in stance after 3-5 steps?

Disregard any intoeing!

Procedure: The child is standing on the side of the examiner and walks toward the parents.

Instruction: Visual by showing what tiptoeing means and verbally by saying; lets see if you can tiptoe all the way to Mummy/Daddy and try to make yourself really long.

If the child starts dropping the heel try to correct it by urging once again to go high up. Observe if child manages to correct him/herself.

**4 points, normal:**

Manages without particular problems. Good ability to roll over to the toes lifts up relatively high and keeps balance. Retains this position throughout the walking path.

**3 points, slightly deviant**:

1. Sufficient power to get up on toes and keeping position throughout the walking path, good upper body alignment though decreased plantar flexion.

Or

1. Appropriate plantar flexion but not enough power to retain position, the heel starts dropping to the ground after a couple of steps.

**2 points, deviant:**

Decreased plantar flexion and insufficient power to retain position, drops heel to the ground with weight shift, can correct but soon drops again.

**1 point, very deviant:**

Clearly decreased active plantar flexion together with compensation on knee/hip level (flexion).

**0 points:**

Cannot accomplish the task.

**4. Heel walking.**

Observe where the child has its center of gravity. Is it perpendicular to the ankle or behind? Is there hyperextension in the knee and or increased hip flexion? How is the position of the foot? Does the foot drop laterally or is there a good balance between invertors/evertors.

COG= centre of gravity.

The child is standing on the parent’s side of the walkway and walks towards the examiner.

Instruction: Visual by showing the child how to walk on heels and saying lets see if you can walk all the way on your heels to me.

Make clear to the child that it is important that he /she lifts his / her feet properly from the floor (show again if necessary).

**4 points, normal:**

Manages without great effort, body position relaxed and the centre of gravity lies over the ankle or slightly shifted backwards. Good ankle dorsal flexion.

**3 points, slightly deviant:**

1. Some effort is needed to keep balance. Decreased dorsal extension, slight compensation such as shifting the COG behind the ankle. Tendency to hyper extend on knee level and flex on hip level.

Or

1. Appropriate forefoot lift though a tendency to drop the lateral border of the foot and or varus of the heel.

**2 points, deviant:**

1. COG clearly behind the ankle joint, clear hyperextension in knee and hip flexion but still good control over the foot.

Or

1. Nearly normal position of COG over the ankle but cannot lift the lateral border sufficient enough from the floor.

**1 point, very deviant:**

There is both highly increased hyperextension on knee level and hip flexion together with varus and drop of lateral border. Compensation mechanism is evident. The child has great difficulties keeping balance.

**0 points:**

Cannot accomplish the task

In the following two parameters both quality and quantity are assessed. Bear in mind the child’s age when assessing performance quality. A child of four does not have the same motor maturity as a child of five and balance maturity may even vary between children of the same age.

The ability for task understanding and in concentrate is an important factor for good performance. Therefore a note should be made about the child’s ability in these factors if deviating from normal.

Definition for balance = keeping the center of gravity within the stance limb surface. Instability is avoided by a shift of the body vector toward the standing limb and strong contraction of the hip abductors to support the pelvis**.**

**5. One leg stance**

Besides determining the quality of performance the number of seconds the child manages to stand on one leg is counted. The best result out of three trials is taken.

Before testing balance on one leg the child should be allowed to have a short rest.

Instruction: Visual by showing and verbally by saying; let see how long time you can manage to stand on one leg. I will be counting one, two, three…..

If the child has problem focusing try to make it look straightforward and concentrate on an object slightly under eye level.

**4 points, Normal:**

The child easily finds its balancing point. Good alignment of upper body over the standing leg. The child looks confident in its position. No significant postural sway.

**3 points, slightly deviant:**

Some effort is needed to find the balancing point. Can keep good alignment with upper body most of the time though (slight postural sway) needs to adjust slightly now and then.

**2 points, deviant:**

The child needs more time to find the balancing point, finds it but soon starts working with upper body and arms to keep balance. Upper body centre of gravity falls outside the foot.

**1 point, very deviant:**

The child needs help to find the balance position. No alignment of upper body. Needs to work a lot with arms and legs to keep standing on one leg.

0 points:

Cannot accomplish the task. None of the criteria above.

**6. Hopping on one leg.**

As well as assessing quality the numbers of hops are also noted for each leg.

Instruction: Visual by showing the child how to hop on one leg and verbal by asking the child to hop as far as possible; How far can you hop!

**4 points, normal:**

Stable body position and alignment. The child seems secure in its task. Finds its balance quickly and hops away with good stride and power. Keeps a straight path.

**3 points, slightly deviant**:

Some effort to find balancing point. Needs some more help from his arms to get going and for maintaining balance.

**2 points, deviant**:

Problem with keeping balance. Difficulty in keeping a straight path. Insufficient propulsion power and/or either short hop stride or unregulated hop stride.

**1 point, very deviant:**

Great difficulties in getting started, finding balance and propulsion upwards/ forwards, manages a couple of hops but falls over.

**0 points:**

Cannot accomplish the task. Cannot even get started.

1. .
